# Differences in the experience of fatigue in patients and healthy controls: patients' descriptions

**DOI:** 10.1186/1477-7525-5-36

**Published:** 2007-07-02

**Authors:** Marieke F Gielissen, Hans Knoop, Petra Servaes, Joke S Kalkman, Marcus J Huibers, Stans Verhagen, Gijs Bleijenberg

**Affiliations:** 1 Expert Centre Chronic Fatigue Nijmegen, Radboud University, Nijmegen Medical Centre, The Netherlands; 2 Department of medical, clinical and experimental psychology, Maastricht University, The Netherlands; 3 Department of Medical Oncology of the Radboud University Nijmegen Medical Centre, The Netherlands

## Abstract

**Background:**

the primary objective was to develop an adjective checklist, the Fatigue Quality List (FQL), aimed at assessing different perceptions of fatigue.

**Methods:**

961 participants filled out the FQL (28 adjectives). A component and confirmatory factor analyses were performed and psychometric properties were evaluated. Differences on factor scores between different patients' groups were investigated and pre- and post treatment scores were compared in demonstrating change of perceptions after treatment of fatigue.

**Results:**

Four independent factors were found with adequate psychometric properties. Different perceptions were found between the patients' groups. Patients who were recovered after treatment for fatigue showed similar scores on the factors as healthy controls.

**Conclusion:**

The FQL appears to be a promising tool in measuring different perceptions of fatigue, which can be especially interesting for clinical practice.

## Background

What is meant by fatigue? Most people are familiar with the experience of fatigue, but the meaning of this sensation can differ between people and even within one person the meaning of fatigue can change. Therefore, fatigue can be defined in different ways and there is no 'gold standard'. Healthy people would characterise fatigue as a pleasant, acute, normal and regulating phenomenon after exercise or a busy day, disappearing after a good night's sleep or a period of rest. However, fatigue can also have a more negative connotation as in fatigue experienced by patients with a health problem. To them fatigue can be a chronic, disabling and life- and activity-limiting experience [1-6].

There are also differences in the factors underlying fatigue severity between patients with different somatic conditions. Processes involved in the experience of fatigue in patients with chronic fatigue syndrome (CFS) are clearly different from processes related to the experience of fatigue in patients with multiple sclerosis (MS) [2] and there are many differences between severely fatigued breast cancer survivors and females with CFS [7].

Because fatigue is not clearly defined, poor communication regarding fatigue exist in the clinical practice [8]. Additionally, health care professionals find consultations on fatigue difficult and are often dissatisfied with or uncertain about the care they provide to patients with fatigue complaints [9,10]. Without appropriate assessment, recognition and providing the proper management to patients with chronic fatigue is difficult. The first necessary step towards improving recognition and management is a thorough understanding of the symptom.

Until now fatigue scales are mostly used to measure fatigue severity [11]. However, fatigue severity does not reflect a persons' perception and appraisal of the fatigue. Therefore, the quantitative way of assessing fatigue fails to capture the nuances and differences in the experience of fatigue. In pain research assessment methods already exists in determining the quality of pain in a patient by using adjectives [12,13].

In this study an adjective checklist, the Fatigue Quality List (FQL), was constructed aimed at assessing different perceptions of fatigue. The development of the FQL was described and additionally three research questions were investigated:

1. Is the FQL a reliable and valid instrument to assess different perceptions of fatigue?

2. Are perceptions of fatigue different between several patient groups with and without chronic fatigue complaints and healthy controls?

3. Do perceptions of fatigue change in patients who recover after treatment for fatigue?

## Methods

### Materials

#### Fatigue Quality List

Researchers and clinicians working at the Expert Centre Chronic Fatigue of the Radboud University Nijmegen Medical Centre made a large list of all possible adjectives that can be used to characterize the feeling of fatigue. The FQL was developed by asking researchers and health care professionals working with patients with unexplained fatigue complaints to indicate on this large list which of the adjectives best fitted with the experience of the fatigue described by their patients. The final list consisted of 28 adjectives most frequently mentioned by the raters.

In filling out the FQL, subjects are instructed to mark with a cross which of the 28 adjectives fit their experienced fatigue. Multiple answers are possible. In this study the Dutch version of the FQL was used. However, the adjectives were translated into English by a back-translation procedure.

*Fatigue severity *was measured by a subscale of the Checklist Individual Strength (CIS-fatigue) consisting of 8 items [14]. Each item was scored on a 7-point Likert scale. High scores indicated a high level of fatigue severity. Based on research with Chronic Fatigue Syndrome patients, a score of 35 or higher on the subscale fatigue severity indicated severe feelings of fatigue. Furthermore, the CIS has excellent psychometric properties [1,11].

### Patients

Nine-hundred-sixty-one participants with a mean age of 43.6 years (sd = 10.2, range 18–79) predominantly female (65%) filled out the FQL. All were either patients or healthy controls participating in scientific studies conducted by the Expert Centre Chronic Fatigue. The total group consisted of:

- 219 cancer survivors. Hundred-twenty-eight (mean age 44.8 (sd = 8.9); female 72%) experienced severe chronic fatigue and 91 (mean age 46.5 (sd = 6.3); female 100%) were not fatigued [3]. Forty-one of these cancer survivors were participating in a randomised control trial about the effectiveness of cognitive behaviour therapy (CBT) especially designed to reduce chronic fatigue in cancer survivors [15]. These patients filled out the FQL at pre- and post treatment.

- 160 patients who were diagnosed with Chronic Fatigue Syndrome (CFS), according to the CDC criteria (mean age 38.0 (sd = 10.7); female 69%) [4,16]. Eighty-two CFS patients who were included in this study were treated for their chronic fatigue complaints with CBT [4]. These patients filled out the FQL two times, at pre- and post treatment.

- 151 employees on sick leave with unexplained fatigue complaints (mean age 44.0 (sd = 8.4); female 55%) [17]. 66 (44%) of these met research criteria for CFS (mean age 42.9 (sd = 8.6); female 61%).

- 276 patients with various neuromuscular disorders. Hundred-sixty-five experienced severe fatigue (mean age 42.2 (sd = 10.6); female 48%) and 120 experienced no fatigue complaints (mean age 42.2 (sd = 11.3); female 48%) [5,18]

- 77 patients who were diagnosed with pancreatitis. Fifty-three experienced severe fatigue (mean age 49.3 (10.0); female 47%) and 24 were not fatigued (mean age 50.2 (sd = 15.5); female 58%).

- 78 healthy persons who experienced no fatigue complaints (mean age 48.2 (6.2); female 100%) [3].

### Statistical analysis

Data analysis was performed using SPSS (version 12.0.1). The total participant group was randomly divided into two groups. A principal component factor analysis was performed in the first group to identify independent factors. A varimax rotation was used to facilitate the interpretation. Furthermore, factor loadings had to be above .40 with a .10 or greater difference in loadings with the other factors. The scree test and the eigenvalues (above 1) were used to identify the number of factors. The factor model was then tested in the second group by using confirmatory factor analyses/AMOS 5.0 (Comparative Fit Index, Goodness of Fit Index, Adjusted Goodness of Fit Index [19,20]).

The internal consistency reliability for each factor was calculated using Cronbach's alpha. Spearman's rho correlations were used to evaluate psychometric properties of the FQL. To investigate the differences between the groups of patients Kruskal-Wallis tests were performed. When the Kruskal-Wallis test was significant, Mann-Whitney-U tests between the groups followed. The sensitivity to change of the FQL was demonstrated by comparing cancer survivors and CFS patients at pre- and post treatment assessment, using the Wilcoxon Signed Ranks Test of matched pairs. To correct for the multiple comparisons, p-value was set on < 0.01.

## Results

### Factor solution

Three of the 28 adjectives were marked with a cross for less than 10% and therefore excluded from further analyses. Final analyses were done with the remaining 25 adjectives. Table 1 presents the final factor solution in the first group (n = 476). Seven adjectives were excluded of factor analysis because factor loadings were < .40 and/or <.10 difference in loadings with the other factors. Both the scree test and eigenvalues indicated a 4-factor solution (Table 2). Factor 1 consisted of 5 adjectives, factor 2 of 4 adjectives, factor 3 of 5 adjectives and factor 4 of 4 adjectives, explaining respectively, 24%, 9%, 6%, 5% of the variance prior to rotation. After rotation the four factors explained respectively, 13%, 12%, 10% and 9% of the variance. Factor 1 was labelled as 'Frustrating', Factor 2 as 'Exhausting', Factor 3 as 'Pleasant' and Factor 4 as 'Frightening'. This four factor model was then tested in the second group (n = 485) by using confirmatory factor analysis. The fit indices indicated an adequate fit. Chi-square (129, n = 485) = 364.5, p < 0.001; Comparative Fit Index = .87; Goodness of Fit Index = .92; Adjusted Goodness of Fit Index = .90.

**Table 1:** Final factor solution: principal-components analysis with varimax-rotation in the first group. Cronbach's Alpha of the four factors

	**Frustrating**	**Exhausting**	**Pleasant**	**Frightening**
discouraging	**.735**			
incessant	**.585**			
annoying	**.680**			
persistent	**.559**			
frustrating	**.704**			
				
exhausting		**.690**		
wearisome		**.537**		
extreme		**.724**		
unbearable		**.509**		
				
temporary			**.400**	
relaxing			**.661**	
fulfilling			**.713**	
normal			**.522**	
pleasant			**.792**	
				
upsetting				**.727**
frightening				**.618**
inexplicable				**.490**
insuperable				**.444**
				
**Cronbach's Alpha**	.79	.68	.61	.57

Three adjectives were excluded of factor analysis because they were marked with a cross for less than 10%: Protective, Soothing, Threatening. Seven adjectives were excluded of factor analysis because factor loadings <.40 and/or <.10 difference in loadings with the other factors: Demanding, Paralysing, Aggravating, Compelling, Treacherous, Insoluble, Acceptable

**Table 2:** principal-components analysis with varimax-rotation, initial eigenvalues

**component**	**Eigenvalues**
1	**4.788**
2	**1.906**
3	**1.285**
4	**1.176**
5	0.984
6	0.875
7	0.813
8	0.742
9	0.714
10	0.664
11	0.623
12	0.593
13	0.568
14	0.514
15	0.503
16	0.445
17	0.428
18	0.380

The four factors were recoded on a 0 to 100 scale, facilitating comparisons between the factors. Higher scores indicate a higher appraisal of the fatigue experience as frustrating, exhausting, pleasant and frightening. The final version of the FQL and the criteria for scoring are presented in appendix A.

### Is the FQL a reliable and valid instrument to assess different perceptions of fatigue?

For each factor the *internal consistency reliability *was calculated in the entire sample of 961 participants, which demonstrated moderate to adequate internal consistencies for all four factors, ranging from .57 to .79 (Table 1).

Supporting *convergent validity *we found that all four factors were statistically significant related to fatigue severity (CIS-fatigue) (Table 3).

**Table 3:** Convergent validity of the 4 factors. Spearman's rho correlation in total group (N = 961)

**Factor**	**Frustrating**	**Exhausting**	**Pleasant**	**Frightening**
Fatigue Severity	.66*	.58*	-.54*	.43*
Exhausting	.54*			
Pleasant	-.48*	-.35*		
Frightening	.49*	.42*	-.25*	
Age	-.16*	-.14*	.05	-.03
Gender (1 = M, 2 = F)	-.09*	.03	.11*	-.10*

* p < 0.01

In calculating *general psychometric properties *statistically significant intercorrelations between the four factors were found (Table 3). Additionally, low correlations were found between the four factors and age and gender, explaining less than 3% of the variance.

### Are the perceptions of fatigue different between several patient groups with and without chronic fatigue complaints and healthy controls?

The non-fatigued groups scored significantly lower on Frustrating, Exhausting and Frightening and significantly higher on Pleasant compared with the fatigued groups (Table 4). The following analyses were performed separately in the fatigued groups and the non-fatigued groups.

**Table 4:** Mean score on 4 factors: comparisons between fatigued disease-free cancer patients, CFS patients, employees with unexplained fatigue, fatigued patients with neuromuscular disease, fatigued patients with pancreatitis, non-fatigued disease-free cancer patients, non-fatigued patients with neuromuscular disease, non-fatigued patients with pancreatitis and healthy persons

	**Frustrating**	**Exhausting**	**Pleasant**	**Frightening**
A. Fatigued disease-free cancer patients	48.6 (30.9)^b,c^	29.3 (28.6)^b,d^	11.7 (17.7)^b,c^	22.7 (24.2)^d,e^
B. Chronic fatigue syndrome	58.5 (32.2)^a,d,e^	37.8 (31.5)^a,c,d,e^	6.6 (13.0)^a,c,d^	25.2 (25.9)^d,e^
C. Employees with unexplained fatigue	63.7 (29.2)^a,d,e^	29.5 (28.1)^b,d^	4.3 (11.2)^a,b,d,e^	26.0 (26.6)^d,e^
D. Fatigued patients with neuromuscular disease	41.8 (32.6)^b,c^	17.8 (24.8)^a,b,c^	13.6 (18.5)^b,c^	13.8 (20.1)^a,b,c^
E. Fatigued patients with pancreatitis	41.1 (33.1)^b,c^	25.9 (29.8)^b^	9.1 (13.9)^c^	14.2 (22.7)^a,b,c^
F. Non-fatigued disease-free cancer patients	8.1 (16.3)	6.6 (14.4)^g^	38.9 (28.3)^g^	7.7 (17.0)
G. Non fatigued patients with neuromuscular disease	9.0 (16.2)	1.7 (7.1)^f,h^	24.7 (21.3)^f,I^	5.6 (13.9)
H. Non-fatigued patients with pancreatitis	13.3 (28.1)	9.4 (17.8)^g^	29.2(23.6)	5.2 (12.7)
I. Healthy persons	7.7 (18.3)	3.9 (13.4)	36.2 (23.3)^g^	3.2 (10.2)

a. significantly different from group A, Mann-Whitney test p < 0.01

b. significantly different from group B, Mann-Whitney test p < 0.01

c. significantly different from group C, Mann-Whitney test p < 0.01

d. significantly different from group D, Mann-Whitney test p < 0.01

e. significantly different from group E, Mann-Whitney test p < 0.01

f. significantly different from group F, Mann-Whitney test p < 0.01

g. significantly different from group G, Mann-Whitney test p < 0.01

h. significantly different from group H, Mann-Whitney test p < 0.01

i. significantly different from group I, Mann-Whitney test p < 0.01

### Frustrating

The non-fatigued groups were similar with respect to the mean scores on Frustrating (p = 0.757). Patients with chronic fatigue syndrome and employees with unexplained fatigue scored significantly higher on Frustrating with respect to the other fatigued groups.

### Exhausting

The non-fatigued patients with various neuromuscular disorders scored significantly lower on Exhausting than the non-fatigued cancer survivors and the non-fatigued patients with pancreatitis. Between the fatigued groups, CFS patients scored significantly higher on Exhausting than the other groups. Furthermore, fatigued patients with pancreatitis scored significantly lower with respect to fatigued cancer survivors and employees with unexplained fatigue. Additionally, patients with neuromuscular disorders scored significantly lower than employees with unexplained fatigue.

### Pleasant

In the non-fatigued group patients with various neuromuscular disorders scored significantly lower on Pleasant than non-fatigued cancer survivors and healthy persons. In the fatigued group employees with unexplained fatigue scored significantly lower on Pleasant than the other groups. CFS patients scored significantly lower than cancer survivors and patients with neuromuscular disorders.

### Frightening

The scores on Frightening in the non-fatigued groups were similar. In the fatigued groups a dichotomy was found between the patients with unexplained fatigue with and without a chronic disease. Fatigued patients without a chronic disease (cancer survivors, CFS patients and employees) scored significantly higher on Frightening than fatigued patients with a chronic disease (patients with a neuromuscular disorder or pancreatitis).

### Do perceptions of fatigue change in patients who recover after treatment for fatigue?

Forty-one fatigued cancer survivors and eighty-two CFS patients were treated for their fatigue complaints with CBT at our department and filled out the FQL at pre- and post treatment. Sensitivity to change of the FQL was demonstrated by dividing the CFS patients and the cancer survivors into two groups: patients who were completely recovered after CBT (CIS-fatigue < 35) and patients who remained fatigued after CBT (CIS-fatigue >= 35). Baseline scores on the four factors were not significantly different between patient who recovered and patients who remained fatigued. The scores on the four factors at pre- and post treatment were compared. Additionally, we compared the post treatment scores on the four factors with the scores of healthy individuals (Table 5). Cancer survivors who were completely recovered after CBT (n = 27) showed a significant decrease on the factors Frustrating, Exhausting and Frightening and a significant increase on the factor Pleasant.

**Table 5:** Comparison of pre- and post treatment scores on the four factors. Comparison of the post treatment scores with those of healthy individuals

			**Frustrating **	**Exhausting **	**Pleasant**	**Frightening**
	**Cancer survivors**					
A	non fatigued after CBT (n = 27)	pre-treatment	52.6 (27.8)	27.8 (24.4)	11.1 (14.0)	22.2 (23.3)
		post-treatment	11.9 (23.0)*	5.6 (20.0)*	36.3 (25.4)*	6.5 (11.2)*
B	still fatigued after CBT (n = 14)	pre-treatment	67.1 (27.9)	46.4 (30.8)	8.6 (17.0)	19.6 (24.4)
		post-treatment	58.6 (34.6)	33.9 (38.7)	7.1 (12.7)	12.5 (19.0)
						
	**Chronic Fatigue Syndrome Patients**					
C	non fatigued after CBT (n = 47	pre-treatment	60.4 (26.5)	42.0 (31.8)	4.7 (8.6)	20.2 (22.5)
		post-treatment	11.1 (18.1)*	3.2 (8.4)*	32.3 (30.5)*	5.9 (11.9)*
D	still fatigued after CBT (n = 35)	pre-treatment	57.7 (30.6)	44.3 (35.9)	5.1 (11.2)	24.3 (24.6)
		post-treatment	45.1 (31.2)	30.0 (33.1)	9.1 (17.0)	12.9 (15.3)*
						
	**Healthy individuals**		7.7 (18.3)^b,d^	3.9 (13.4)^b,d^	36.2 (23.3)^b,d^	3.2 (10.2)^b,d^

CBT = cognitive behaviour therapy

* significant difference between pre- and post treatment scores, Wilcoxon signed rank test p < 0.01

a. significantly different from post treatment scores of group A, Mann-Whitney-U test p < 0.01

b. significantly different from post treatment scores of group B, Mann-Whitney-U test p < 0.01

c. significantly different from post treatment scores of group C, Mann-Whitney-U test p < 0.01

d. significantly different from post treatment scores of group D, Mann-Whitney-U test p < 0.01

The post-treatment scores were not significantly different from those of healthy individuals. In contrast, the cancer survivors who still remained fatigued after CBT (n = 14) did not show a change in the scores on the four factors from pre- to post treatment. Furthermore, their scores at post treatment were significantly different from the scores of healthy individuals. In investigating CFS patients who recovered after CBT (n = 47) the same pattern was found. They also decreased significantly on the factors Frustrating, Exhausting and Frightening and increased significantly on the factor Pleasant. The scores at post treatment were not significantly different from those of healthy individuals. CFS patients who were not recovered after CBT (n = 35) showed no change between pre- and post treatment scores on the factors Frustrating, Exhausting and Pleasant. Although a significant decrease was seen on the factor Frightening, the post treatment scores of the four factors were significantly different form those of healthy individuals.

## Discussion

The present study shows that the FQL provides a self report instrument that assesses the perceptions of fatigue. The FQL consists of four coherent factors, namely Frustrating, Exhausting, Pleasant and Frightening. The stable pattern of these factors was indicated with a confirmatory factor analyses, revealing an invariant internal structure in a second group of patients. Furthermore, the data of this study show that the FQL has adequate psychometric properties. Both the intercorrelations and the correlations of the four factors with the subscale CIS-fatigue were not to the extent that the factors could be seen as a parallel test, thus supporting the relative uniqueness of each factor.

The assumption that fatigue is experienced differently by everybody is confirmed with the data of this study. Severely fatigued patients had different perceptions of fatigue compared to healthy individuals. The healthy persons described fatigue as temporary, relaxing, fulfilling, normal and pleasant. None of these adjectives were chosen by 70% of the severely fatigued patients. Even patients with similar fatigue severity, appreciated fatigued differently. Different patterns were seen on the four factors of the FQL between the different populations of patients experiencing fatigue. CFS patients and severely fatigued employees had the highest score on the factors Frustrating, Exhausting and Frightening and also the lowest score on the factor Pleasant in contrast with the other fatigued groups. Until now the underlying aetiology of CFS still remains unclear [21,22]. Because the patients can not attribute their fatigue to a distinct cause, it's possible that they are more focussed on their fatigue and perceive their fatigue in a more negative way, than the other groups. In agreement with this finding, Moss-Morris et al. [23] found that CFS patients had a more negative view about their symptoms than patients with Rheumatoid arthritis (RA). Additionally, Taillefer et al. [24] found higher levels of illness worry in CFS patients than MS patients who were fatigued. Results of the FQL also showed that patients with a current chronic disease experience their fatigue as less frightening than patients with no current or a past disease. It is possible that these patients attribute their fatigue to their illness and therefore perceive it as less frightening. Cancer survivors may experience fatigue as highly anxiety provoking because they can see fatigue as a symptom for disease-recurrence. Therefore fatigue can be labelled as frightening [25]. Future research is necessary to examine if the FQL is applicable for individual assessment and furthermore investigate what the effect of these different perceptions is on the management of fatigue complaints in the clinical practice.

To reach recovery not only a decrease in fatigue severity is important, it is also important that a change in the evaluation of fatigue in the patient occurs. As fatigue is also a part of normal health, being recovered also includes feeling tired sometimes. This makes it difficult to decide where experiencing fatigue as a sign of illness ends and the experience of normal health surfaces. During CBT patients learn that fatigue may occur as part of normal healthy life. When a decrease is seen in the fatigue severity of a patient and the evaluation of the fatigue stays negative, it implicates that a patients still suffers and is disabled due to the fatigue. The patient cannot be seen as fully recovered [26]. The results of this study showed that the FQL can demonstrate change in fatigue perceptions following treatment of fatigue. Patients who were recovered after CBT had the same scores on all four factors compared to healthy persons. So, not only the fatigue severity changed after therapy but also the evaluation of fatigue. The FQL can therefore be a helpful tool to define full recovery in the clinical practice.

## Competing interests

The author(s) declare that they have no competing interests.

## Authors' contributions

MG contributed to conception and design, data collection, data analysis and interpretation, drafting the manuscript, writing the manuscript. HK contributed to data collection, was involved in drafting the manuscript and revising the manuscript. PS contributed to data collection and was involved in drafting the manuscript and revising the manuscript. JK contributed to data collection and revising the manuscript. MH contributed to data collection and revising the manuscript. CV contributed to interpretation of the data and revising the manuscript. GB was involved in the conception and design, drafting the manuscript, interpretation of the data and revising the manuscript. All authors have given approval to the version to be published

## Appendix A

### Fatigue quality list – FQL

Fatigue can be described in different ways. (see table 6 for form)

**Table 6:** Fatigue Quality List

Fatigue can be described in different ways. The adjectives below can be seen as descriptions of fatigue.	
Please indicate which adjectives accurately describe the fatigue you experienced during the last two weeks by marking them with a cross.	
upsetting	persistent
discouraging	frustrating
temporary	relaxing
exhausting	inexplicable
incessant	fulfilling
wearisome	insuperable
frightening	unbearable
annoying	normal
extreme	pleasant

#### Scoring FQL

Subsequently the four factors are calculated by summing the respective items (0 – 100):    

Factor 1: Frustrating 

Score of each item: 20  

Adjectives: discouraging, incessant, annoying, persistent, frustrating    

Factor 2: Exhausting

Score of each item: 25  

Adjectives: exhausting, wearisome, extreme, unbearable    

Factor 3: Pleasant 

Score of each item: 20  

Adjectives: temporary, relaxing, fulfilling, normal, pleasant    

Factor 4: Frightening

Score of each item: 25  

Adjectives: upsetting, frightening, inexplicable, insuperable  
